# Editorial: Post-anesthesia Cognitive Dysfunction: How, When and Why

**DOI:** 10.3389/fnbeh.2021.797483

**Published:** 2021-11-25

**Authors:** Ana M. Valentim, Stefano Gaburro, Matthew O. Parker

**Affiliations:** ^1^Laboratory Animal Science Group, Instituto de Biologia Molecular e Celular, Universidade do Porto, Porto, Portugal; ^2^i3S—Instituto de Investigação e Inovação em Saúde, Universidade do Porto, Porto, Portugal; ^3^Tecniplast S.p.A., Buguggiate, Italy; ^4^Brain and Behaviour Lab, School of Pharmacy and Biomedical Sciences, University of Portsmouth, Portsmouth, United Kingdom; ^5^The International Zebrafish Neuroscience Research Consortium, Slidell, LA, United States

**Keywords:** anesthesia, cognitive dysfunction, working memory, EEG, pediatric intensive care, fMRI, LMR

Anesthetics are used daily in hospitals and in clinics, but also in research laboratories to induce anesthesia, analgesia, and sedation to millions of humans and non-human animals. The processes by which these agents act in the brain is still not fully understood. There is a bulk of research claiming that this process may not be fully reversible causing short- or long-term effects on neuronal functions. Some papers have contradictory results as this process depends on several variables, including the anesthetic type and its concentration, the duration of anesthesia, the type of cognitive test, the genetic profile, age, and health status of the subject (Belrose and Noppens, 2019). Much of the research in this area comprises laboratory animal models rather than humans because it is very hard to control extraneous variables in clinical settings (Leung and Sands, 2009). Therefore, care must be taken when translating these findings from animal models to humans. However, knowing more about the mechanisms of anesthesia and their side-effects is not only crucial to identify the best anesthetic protocols, but also to prevent the risks associated to each situation and patient.

The aim of this Research Topic was primarily to shed light on potential cognitive impairments caused by the use of anesthetics in humans and non-animals, in both clinical and pre-clinical research settings. The authors in the topic have provided data and scholarly reviews which will increase the knowledge of the field. The contributions include: novel methods to evaluate or predict cognitive dysfunction; enhancing the importance of sleep and EEG analysis; the implications of using anesthesia in research; and the urgency to understand the impact of anesthetics' use in critically ill patients with different ages.

The use of animal models in anesthesia research has typically been limited to mammals. However, fish are vertebrates with well-conserved hypnotic (Renier et al., 2007) and cognitive (Best and Alderton, 2008) pathways. To highlight the potential utility of zebrafish in this area of research (Felix et al., 2019), Fontana et al. raise awareness regarding the potential problems that anesthesia may cause when used in research. In their paper, Fontana et al. confirmed that zebrafish anesthetized with tricaine (the most used anesthetic in zebrafish) showed changes in working memory and cognitive flexibility for 2 days following treatment. A potential alteration on working memory caused by anesthesia was also debated in Manzella et al., who point out that although the impact of anesthesia in neonatal animals has been widely reported, the study of long-term alterations on neuronal oscillations has received little attention. Normal neuronal oscillatory patterns are important for plasticity, memory consolidation, and sleep. Manzella et al. showed that neonatal exposure of rats to isoflurane caused extensive neurotoxicity but did not disrupt sleep architecture in adolescent rats. However, the use of more than one anesthetic, as is often the case in surgery or other management procedures, may induce sleep disruption (Lunardi et al., 2019). This neonatal exposure of isoflurane caused a reduction in beta oscillations, more specifically in beta 1 range, associated with wake behavior. Based on the way beta 1 (around 15 Hz) oscillations work, Manzella et al. suggested that beta 1 plays a role in working memory, thus suggesting a mechanism through which isoflurane may disrupt working memory of adolescent rats.

Concern regarding the younger population and sleep disruption is also projected in the work of Turner et al. Their paper stressed that more research is needed in critical ill patients, as the anesthetics and analgesics are not only used in surgeries but also in ICU for sedation, and pain management. They argue that translational research with animal models should also have this in consideration. Also, the management strategies used for pediatrics must be different to adult critical illness, due to lower resilience in childhood and vulnerability during early neurodevelopmental periods. Both analgesia and sedation has been identified as risk factors for delirium and neurological sequalae. Sedation can also induce sleep disruption, which is often disregarded but contributes to cognitive sequelae. In fact, some studies have highlighted that EEG and polysomnography reveal alterations in sleep architecture (Armour et al., 2011). In the end, Turner et al. proposed strategies to improve sleep quality, and stressed the advantage of cognitive rehabilitation program supported by cognitive experts in collaboration with Pediatric Intensive Care Unit workers.

In addition to managing cognitive dysfunction, it is essential to have tools to predict and/or to make a prognosis, especially because the impact of anesthesia can be very variable between individuals. In an opinion article, Aksenov discussed the potential to use resting state functional MRI (rsfMRI) as a diagnostic and prognostic for anesthesia-induced neuronal deficiency in young brains. Anesthesia acts on neurotransmitters, potentially interfering with the development of glutamatergic and GABAergic system; thus, the development of vasomotion can also be affected and observed with rsfMRI. In fact, a study showed deficiency in short-range rsfMRI connectivity in adult animals after neonatal anesthesia exposure (Drobyshevsky et al., 2020). Aksenov hypothesized that, if neuronal apoptosis induced by neonatal anesthesia leads to neurovascular deficiency, this can be studied using rsfMRI based on the markers of frequency, amplitude, periodicity, or synchrony of arteriolar vasomotion.

The influence of inflammation on the post-operative cognitive dysfunction (POCD) has been widely reported (Peng et al., 2013). Thus, Zhao et al. proposed a novel predictor of cognitive dysfunction after cardiovascular surgery in older individuals (more than 50 years): the lymphocyte-to-monocyte ratio (LMR). This ratio has been used as a prognostic marker in a variety of cardiovascular diseases, and neurodegenerative diseases (Umehara et al., 2020). According to their results, LMR has potential to be a preoperative biomarker for POCD, as high-LMR increased the risk of the occurrence of POCD. This could be extremely useful for the surgical team to manage the perioperative risk factors depending on the LMR value of each patient.

## Author Contributions

AV wrote the first draft of the manuscript. SG and MP contributed to manuscript revision, read, and all authors approved the submitted version.

## Funding

This work has been funded by FEDER funds through COMPETE 2020 and the POCI (POCI-01-0145-FEDER-029542), Portugal 2020, and by Fundação para a Ciência e a Tecnologia under the project PTDC/CVT-CVT/29542/2017.

## Conflict of Interest

SG is employed by the company Tecniplast S.p.A. The remaining authors declare that the research was conducted in the absence of any commercial or financial relationships that could be construed as a potential conflict of interest.

## Publisher's Note

All claims expressed in this article are solely those of the authors and do not necessarily represent those of their affiliated organizations, or those of the publisher, the editors and the reviewers. Any product that may be evaluated in this article, or claim that may be made by its manufacturer, is not guaranteed or endorsed by the publisher.
